# The Risk of Fatal Arrhythmias Associated With Sertraline in Patients With Post-myocardial Infarction Depression

**DOI:** 10.7759/cureus.28946

**Published:** 2022-09-08

**Authors:** Sai Dheeraj Gutlapalli, Keerthana Prakash, Kiran Maee Swarnakari, Meena Bai, Mohana Priya Manoharan, Rabab Raja, Aneeque Jamil, Denise Csendes, Aditya Desai, Darshi M Desai, Michael Alfonso

**Affiliations:** 1 Internal Medicine, California Institute of Behavioral Neurosciences & Psychology, Fairfield, USA; 2 Medicine, California Institute of Behavioral Neurosciences & Psychology, Fairfield, USA; 3 Internal Medicine/Clinical Research, California Institute of Behavioral Neurosciences & Psychology, Fairfield, USA

**Keywords:** mental health illness, cardiovascular disease, ventricular tachycardia (vt), life threatening arrhythmia, depression, post-myocardial infarction, sertraline

## Abstract

Sertraline is a first-line antidepressant and the most commonly used in the treatment of selective serotonin reuptake inhibitor (SSRI) in major depression. It is preferred due to its central and peripheral actions on the serotonergic system in patients with mental health issues as well as cardiovascular disease, particularly post-myocardial infarction depression. Some of the feared adverse effects include QT prolongation, arrhythmias including Torsades de pointed, and sudden cardiac death, which are associated with older antidepressants and are rarely seen with SSRIs, including sertraline. We tried to understand the risks associated with sertraline use in cardiac patients. We reviewed all the relevant information from inception up to July 2022 regarding the risks of sertraline use in cardiovascular diseases, particularly with a focus on post-myocardial infarction depression, and gathered around 500 articles in our research and narrowed it down to 37 relevant articles. The database used was PubMed and the keywords used are sertraline, arrhythmia, major depression, post-myocardial infarction, and ventricular tachycardia. We carefully screened all relevant articles and found articles supporting and refuting the effects of sertraline in increasing cardiovascular morbidity and mortality. We concluded that there is a significant variability due to confounding factors affecting individual cases. Overall, sertraline has no increased risk in comparison with other antidepressants and a comparatively preferable safety profile to other SSRIs like citalopram in general cases. Any patient with a high risk of arrhythmias due to any etiology should receive a screening ECG before sertraline prescription for baseline QT interval and genotyping for any serotonin transporter/receptor variations. Patients should also be periodically monitored for drug-drug interactions while on therapy. We encourage further research, including randomized clinical trials and post-marketing surveillance regarding the use of sertraline in high-risk cases.

## Introduction and background

Cardiovascular disease (CVD) is frequently associated with major depression (MD); it may precipitate myocardial infarction (MI) or may be triggered by it and vice versa [1]. Approximately twenty-five thousand people with CVD die every day in the United States [2]. Coronary artery disease (CAD) is linked to the incidence of psychological disorders such as anxiety, panic attacks, and major depressive disorder (MDD), with a high frequency of association and multi-directional causality [3-5]. Factors like depression, anxiety, stress, and lower socioeconomic strata are directly linked to a worse prognosis with pre-existing CAD [6]. Depressive symptoms are prevalent in the majority of CAD patients, and 20-35% have major depression [7]. The risk of morbidity and mortality in CVD is independently linked to major depression [8].

Selective serotonin reuptake inhibitors (SSRIs) like sertraline are the preferred pharmacological treatments in CVD patients due to minimal cardiac side effects; electrocardiogram (ECG) monitoring is still advisable in high-risk patients to screen for potential QT prolongation [3]. SSRIs have similar efficacy to older antidepressants like tricyclic antidepressants (TCAs) but have a much more favorable profile of adverse effects [9]. Anxiety and depression are linked to medication non-compliance and reduced treatment follow-up, increased risk of hospitalizations, reduced overall function, and high mortality in heart failure (HF) [10]. Evidence shows an improvement in long-term survival in patients with CAD and co-morbid depression who received SSRIs, the majority of patients achieved remission in two months on SSRI monotherapy [5]. Depression is an important predictor of new-onset and recurrent atrial fibrillation; anxiety and stress are also known as arrhythmia triggers [6]. When prescribing SSRIs to patients with CVD, especially arrhythmias, HF, and MI, we must carefully consider the risk of QT prolongation and drug-drug interactions [6]. Even in patients without a history of CVD, there is an increased risk of arrhythmia and sudden cardiac death (SCD) associated with depression [7]. The cardiac adverse effect profile of antidepressants ranges from hypotension, hypertension (HTN), bradycardia, tachycardia, electrolyte imbalances, ECG abnormalities, reduced cardiac conduction and output, arrhythmias, and SCD [7]. Current evidence supports the use of cognitive behavior therapy (CBT) and psychotherapy along with pharmacotherapy for the treatment of depression in CVD patients [11].

Our research focuses on the cardiovascular adverse effects of sertraline in post-MI depression. In view of the immense global burden of major depression in CVD patients, we try to understand the risks and benefits of sertraline treatment by focusing on cardiovascular morbidity and mortality. The relevant data for this literature review was gathered from the PubMed database; five keywords were used "arrhythmia", "major depression", "post-myocardial infarction", "sertraline", and "ventricular tachycardia", and the search was performed using medical subject headings (MeSH) strategy. We have manually screened and included all the relevant articles we could find since inception till July 20, 2022. All data is sourced from PubMed.

## Review

Depression and the heart

In the coming decade, depression may become the single largest contributor to medical illness around the world [8]. Heart disease is the leading cause of death on the planet, while CAD and depression are the major issues leading to long-term disability [5,7]. SSRIs are the most frequently used antidepressants among the more than twenty different drugs available around the world in the last few decades [9]. In patients with pre-existing CVD, there is a higher risk and frequency of mental illness like anxiety, depression, and panic disorders leading to worsened prognosis post cardiac events, and patients with mental health issues are at increased risk of new-onset CVD; MDD can trigger MI and vice versa, almost half of CVD patients have low mood and depressive symptoms while 20% have MDD [1,3,4,6,7]. Cardiovascular disease and mental health disorders have a multi-directional association [3,5]. Almost 65% of MI patients have an associated psychiatric illness, usually depression and anxiety. Major depression is observed in at least 40% of CAD patients in their lifetime. Major depression is reported in 25% of patients post-coronary artery bypass graft (CABG) surgery. History of depression increases the risk of acute MI by four times compared to controls in a study, and a history of MI is an independent risk factor for new-onset depression in hospital; patients with depression in the hospital had a higher incidence of depressive symptoms after discharge [5]. An 18% higher risk of developing HF and accelerated progression of pre-existing HF was associated with depression over a seven-year follow-up in various studies [10].

**Figure 1:**
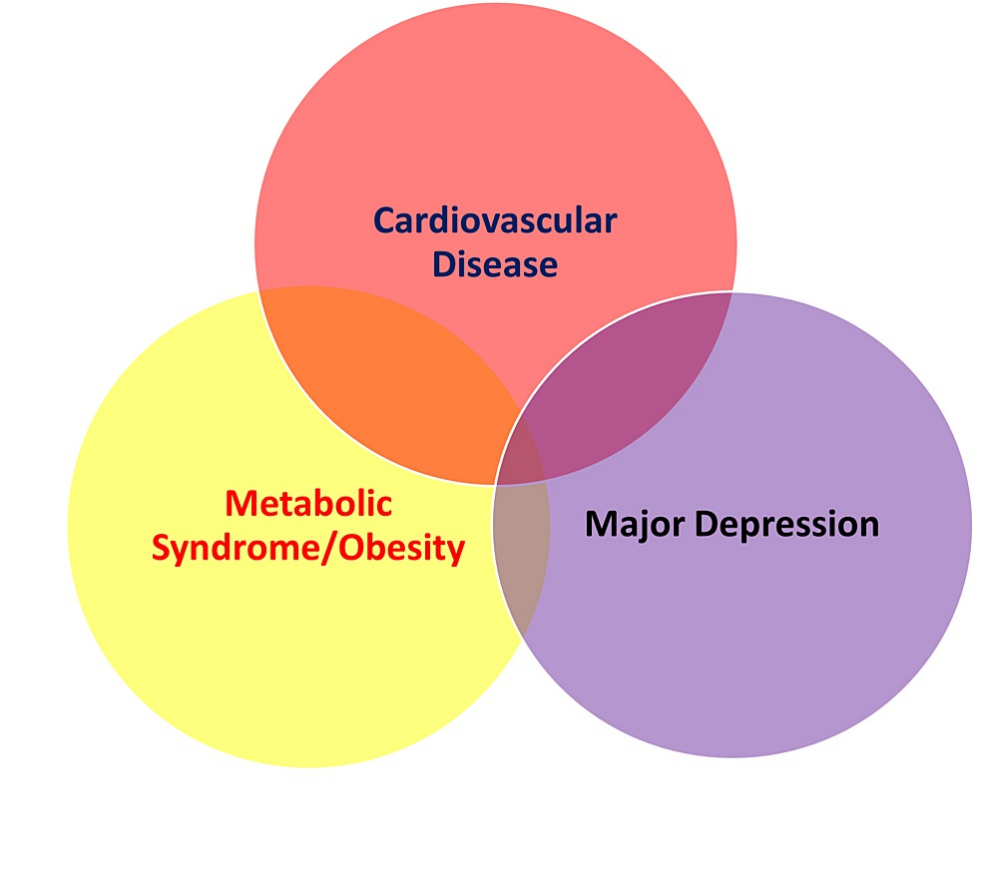
Overlapping pathological spheres of cardiovascular disease, major depression, obesity and metabolic syndrome

Mental health disorders are associated with three times higher risk of medication non-compliance, increased risk of diabetes, malnutrition, smoking, obesity, alcohol abuse, sleep disorders, substance abuse, frequent hospitalizations, and higher mortality in CVD patients [5,6,10,11]. Antidepressant efficacy is reduced in patients with anxiety [10]. Depression promotes inflammation, increasing the risk of negative outcomes in CVD and psychiatric illness by promoting fibrosis and adverse ventricular remodeling. Heart rate variability (HRV) is reduced in MDD along with autonomic dysregulation increasing arrhythmic risk and promoting adverse cardiac remodeling [10]. Endothelial dysfunction, elevated C-reactive protein (CRP), platelet dysfunction, and reduced flow-mediated vascular dilation leading to accelerated atherosclerotic plaque formation in CAD patients are linked with depression [5,10]. A prodrome of minor depression often precedes MI by four to five years [5]. Higher rates of angina, recurrent MI, arrhythmias, CHF during the initial hospital stay and higher rates of readmissions are seen in depressed patients in comparison to non-depressed individuals; CVD morbidity and mortality were doubled in depression; depression is an important predictor of mortality post-MI and CHF hospitalizations [5,8]. Type D personality is linked to a higher risk of depression and anxiety [6]. Arrhythmia and SCD risk are higher in depression, even in the absence of CVD [7]. In America, over eight million people were diagnosed with HF until 2020, with a 50% mortality risk within the subsequent five years, and four million of these patients have depression as a comorbid condition [6,7,10]. The overlapping association between CVD, MDD, obesity, and metabolic syndrome is shown in Figure 1.

The American Heart Association states that MDD risk is two to three times higher than the general population in patients with acute coronary syndrome (ACS) and vice versa; prognosis is further worsened by associated serotonin, dopamine, norepinephrine dysregulation, sympathetic and parasympathetic dysfunction in ACS with comorbid MDD [7,8]. Baroreflex sensitivity (BRS) and heart rate variability (HRV) are essential tools to understand these mechanisms [8]. HRV is a good predictor of arrhythmias risk along with ECG markers; a normal ECG does not preclude arrhythmias; therefore, HRV is a useful method for arrhythmic risk stratification in certain patients. A high HRV is protective against the development of HF, MI, and fatal arrhythmias [7]. Cardiovagal activity dictates HRV and 70% of cardiovagal tone is under the influence of the arterial baroreflex system. Acute stress, HTN, dilated cardiomyopathy, chronic CHF, MI, the incidence of ventricular fibrillation after MI, and increased risk of SCD are all associated with reduced BRS and HRV [8]. In the U.S., 6.6 million Americans and fifteen million people in Europe are currently diagnosed with CHF. Depression triples the risk of CHF and doubles mortality compared to the general population, and stifles proper treatment [11].

In America, 15% of the elderly are taking antidepressant medication [12]. The lifetime prevalence of depression is around 20% globally [13]. ACS is a broader term for unstable angina (UA) and MI. ACS affects a million Americans every year; half of them are previously depressed. Anxiety is independently associated with CVD, and 25% of CVD patients have a generalized anxiety disorder (GAD). Half the patients post-MI have acute anxiety, with many having persistent symptoms for up to two years. The incidence of anxiety is 40% higher in patients with CAD than in those without CAD. Untreated depression has been observed to be stable for years post-MI. Patients with major depression and ACS in-hospital had been reported to be depressed for more than four weeks in 95% of the cases, and 60% were depressed for more than six months [14].

High levels of corticotropin-releasing factor (CRF) in cerebrospinal fluid (CSF) were observed in depressed patients leading to rising in corticosteroids, accelerating atherosclerosis, hypertriglyceridemia, HTN, and hypercholesterolemia [5]. Major depression increases QT variability and decreases HRV leading to elevated arrhythmia risk; lower HRV increases mortality risk post-MI [5,7]. Stress levels during childhood and adolescence are linked to the development of CVD later in life [6]. Social support is protective for the prevention and progression of CVD [6]. Four to five times higher death rate was observed in socially isolated and highly stressed males during acute MI and post-MI [15]. Cognitive deficits are present in half the patients with cardiac illness, especially in patients having severe cardiovascular insufficiency or a history of open heart surgery, and the risk of dementia is doubled in HF [6]. Patients who develop post-traumatic stress disorder (PTSD) after MI and depressed CVD patients, in general, should be screened for comorbid dementia [6,13].

Acute psychological stress is a known trigger for ventricular tachycardia (VT), stress-induced cardiomyopathy, and ACS. Significant impairment in quality of life is seen with supraventricular tachycardia (SVT) and sometimes can be confused with panic attacks in patients with anxiety disorders. Stress, anxiety, and depression are known to trigger arrhythmias, and MDD is a predictor of new and recurrent atrial fibrillation. Functional heart problems like palpitations and chest pain without an organic cause confound clinicians as they are mainly psychogenic in nature in almost 15% of cases, and more than half of functional heart problems are seen in patients with mental health issues [6].

**Figure 2:**
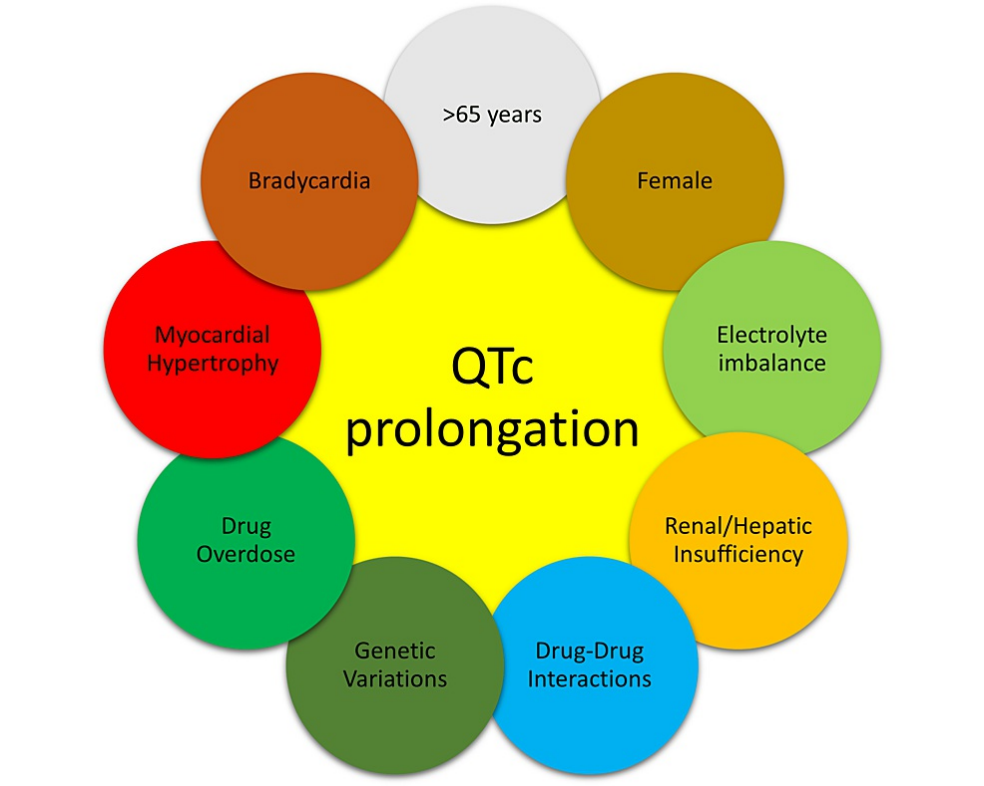
The factors affecting QT interval prolongation

The QT interval in ECG is an important marker for the development of arrhythmias, A normal corrected QT interval (QTc) length is approximately 400 milliseconds (ms), and QTc exceeding 500 ms increases the risk of Torsades de pointes (TdP), a dangerous and potentially fatal polymorphic ventricular tachyarrhythmia and SCD. The important risk factors for QTc prolongation are age >65 years, female gender, bradycardia, myocardial hypertrophy, hypomagnesemia, hypokalemia, pre-existing heart disease, drug overdose, reduced drug clearance due to renal/hepatic failure, genetic ion channel polymorphisms, variations in cytochrome enzymes reducing drug metabolism and congenital long QT syndromes [7]. The various factors affecting QTc prolongation are shown in Figure 2.

Post-MI depression is correlated with the level of cardiac dysfunction and the likelihood of progression to CHF [11]. A high rate of depression was linked to reduced left ventricular ejection fraction (LVEF) 3-12 months post-MI, and LVEF <30% post-MI has a 4.46 higher odds ratio for the incidence of depression compared to patients with LVEF >60% (preserved EF). The most effective treatment for depression for the majority of patients is electroconvulsive therapy (ECT) which has a response rate four times higher than pharmacotherapy, "cardiac-modified ECT protocol" is ideal in patients with cardiac disease [11]. Roughly $50 billion is spent every year on the medical costs associated with CHF alone, with a further 30% higher cost of care for patients with comorbid depression in America; the cost of care for depression is 1% of the overall GDP of Europe [8,11]. According to the Johns Hopkins precursor study, depression is an independent risk factor for the incidence of CVD and MI in the long term; depression increased CVD risk by 60% [14]. Depression pre- and post-MI significantly worsened medical outcomes in comparison to non-depressed patients with a higher incidence of recurrent adverse events in-hospital and mortality. Studies show that post-MI depression increased the risk of mortality by 2.6 times at one-year follow-up and doubled the risk of recurrent MI compared to non-depressed patients [13]. The highest risk of adverse outcomes was associated with treatment-resistant post-MI depression [14]. Patients with MDD may have readmission rates as high as 80% in one-year post cardiac events [16].

Severe anxiety is a stronger risk factor for heart disease than depression. Anxiety is linked with significantly higher short- and long-term morbidity and mortality during acute CVD events and post-MI [13]. Patients with post-MI depression with HF have lower motivation to follow a healthy lifestyle and complete cardiac rehabilitation [9,13]. Patients with post-MI depression have a two to three times higher risk of mortality from cardiac and non-cardiac causes [2]. Thirty minutes of daily exercise have similar effectiveness as sertraline against depression and reduce all-cause mortality by 25% in CVD. Post-MI depression during cardiac rehabilitation is effectively treated by a combination of psychotherapy, antidepressants, and exercise with a 75% reduction in mortality compared to patients who did not exercise; regular exercise alone reduced non-fatal reinfarction, adverse event, and overall mortality by 50% post-MI at two years follow-up [5,6,13,14]. Depression and anxiety in HF are effectively treated by cognitive behavior therapy (CBT) with improvement in quality of life post-MI; antidepressants are more effective in CVDs, especially CAD [5,10,14,17].

Globally 30% of deaths yearly are due to heart disease, and 30% of CAD or HF patients have anxiety/depression. Anxiety/depression during and post-ACS worsen the risk of adverse events in-hospital and long-term cardiac morbidity and mortality. Anxiety and depression lead to noradrenergic hyperactivity and serotonergic dysregulation, causing poor outcomes by means of vascular stress caused by catecholamines on blood vessels, platelet disequilibrium, inflammation, lower HRV, endothelial dysfunction, and cardiac remodeling, worsening prognosis of CAD, ACS, and MI [5,10,14,17]. A study on the effectiveness of CBT and religious cognitive behavior therapy (RCBT) showed that over twelve sessions of therapy, RCBT was slightly more effective in religiously inclined people, but either RCBT or CBT was effective in treating high-risk CVD patients with MDD [18].

Serotonin in cardiovascular disease

Depressed patients with MI have altered biological responses to serotonin regulation in both brain and peripheral tissues [15]. Serotonergic transmission, both centrally and peripherally, is a common link between CVD and MDD [19]. High incidence of MI is seen in patients with a history of MDD; this association is due to dysregulation of the serotonin transporter (SERT), causing increased activation of platelets in depression, leading to a higher risk of coagulopathy and coronary arterial obstruction, platelets have the largest concentration of serotonin in the body and cannot synthesize their own serotonin [15]. Platelet serotonin is entirely due to uptake, and SSRIs may negatively regulate this process [15]. Intracellular serotonin is necessary for the production of cytokines and drugs that affect serotonin levels, as well as polymorphism in serotonin receptors, which significantly affect the production of inflammatory markers [19].

Depression leads to an increase in proinflammatory cytokines, tumor necrosis factor-alpha (TNF-α), interleukin-1 (IL-1), and interleukin-6 (IL-6) which stimulate central serotonin neurotransmission and are linked to hypothalamic-pituitary-adrenal overactivity increasing the risk of CVD and MI. IL-6 gene polymorphism is linked to a lower risk of depression with interferon therapy. Proinflammatory cytokines like IL-1, IL-6, and TNF are linked to the incidence and progression of HF, leading to ventricular dilation and myocardial dysfunction days to weeks after MI. Proinflammatory cytokines are also responsible for the myocardial response to injury and help in maintaining homeostasis through induction of myocardial tissue repair; they play an adaptive role in the early stages of HF. TNF-α is released from the ischemic myocardium after acute MI and promotes the release of other cytokines like IL-1 and IL-6 leading to inflammation and cell adhesion needed for repair. Polymorphism of IL-6 174 G/C is linked with an increased risk of MI [19]. Pleiotropic expression is observed with serotonin transporter gene SLC6A4 with two major polymorphisms, the S allele, and the L allele; S allele heterozygotes expresses half as much SERT as compared to LL homozygotes [20]. 5-HTTLPR polymorphism of S allele may attenuate response in MDD to SSRIs. Homozygotes for S allele were three times lower rates of remission compared to patients with other genotypes. Serotonin-transporter-linked promoter region (5-HTTLPR) polymorphisms are associated with the integrity of white matter and influence neural development throughout life. In an analysis of depressed elderly patients who were genotyped, patients with lower fractional anisotropy were more likely to have depression, and the S allele genotype is associated with a lower likelihood of MDD remission. Depressed elderly patients with low SERT expressing 5-HTTLPR alleles had more white matter abnormalities and difficulty achieving MDD remission [20].

**Figure 3:**
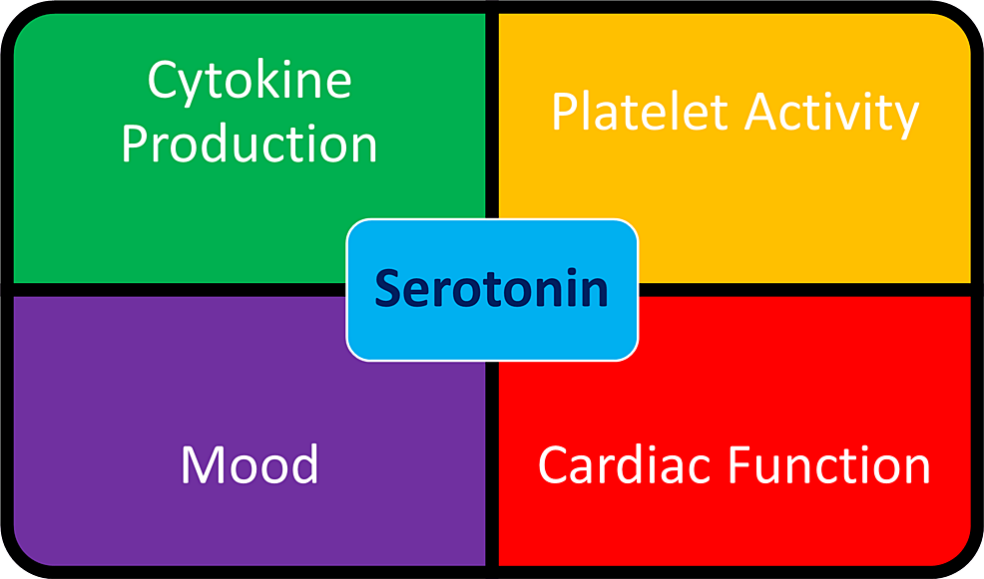
Important effects of serotonin

Serotonin signaling in the brain is an important regulator of neurogenesis in adults and is influenced by SERT. Reduced SERT expressing 5-HTTLPR alleles directly account for about 30% variation in depression severity and the presence of S allele increased the risk of depression due to stress; the S allele increased vulnerability to hypothalamic-pituitary axis (HPA) axis hyperactivation, leading to impairment in memory and decreased hippocampal volume. Some 5-HTTLPR polymorphisms are closely linked to a serious risk of vascular disease; SL allele causes increased triglycerides and cholesterol levels, directly leading to a greater risk of heart disease, angina, and MI in elderly patients. Patients with S allele, including carriers, when compared to homozygotes for L allele, had a significantly higher risk of cardiac events like SCD, arrhythmia, unstable angina, recurrent MI, and HF after an acute MI and was partly mediated by symptoms of depression. Sensitivity to hypercholesterolemia was increased in depressive episodes in S allele carriers [20]. Lower levels of dehydroepiandrosterone (DHEA) and higher levels of inflammatory cytokines are reported in patients with CAD and MDD [18]. The important effects of serotonin are shown in Figure 3.

Sertraline and arrhythmias

To cause an arrhythmia, it requires a susceptible myocardium and an inciting incident/trigger; for example, a scar from prior MI may increase susceptibility, and a trigger like ventricular premature complex can incite arrhythmic events. Somatic symptoms associated with arrhythmias may cause depression, and reverse causality may also be seen. Anger is also an important trigger for arrhythmia [21]. Testosterone is protective against QTc prolongation, while estrogen increases the risk of QTc prolongation [22]. Increased risk of cardiac arrest is seen in patients with depression even with the use of antidepressants; a study that included HTN, DM, and CHF during follow-up for eight years reported an increased risk of SCD in patients with depression [21].

The major link between anger, anxiety and emotional states with increased risk of arrhythmia and SCD is postulated as sympathetic overactivity and parasympathetic suppression. QT variability index was significantly higher in depression and is a marker for ventricular arrhythmias. Profound catecholamine surge is an important cause of stress cardiomyopathy and may be associated with anxiety, emotional distress, and depression. SCD is more common in long QT syndrome type 2 due to emotional distress. In contrast to ventricular arrhythmias, parasympathetic stimulation may increase the risk of atrial fibrillation [21].

When considering the upstream and downstream therapies for patients with post-MI depression, which may decrease arrhythmia risk, most of them involve regulating the autonomic nervous system; the most important such therapy is the ICD, which is mainly limited by the associated financial burden and psychological distress [21]. It is hypothesized that SSRIs inhibit the progression of atherosclerosis and decrease the risk of cardiac complications like arrhythmias, platelet hyperactivity, proinflammatory markers, cortisol levels, and positively affect HDL levels [23]. In regard to SSRIs, QTc prolongation has mostly been associated with citalopram therapy and is very rarely associated with sertraline [24]. Studies have shown that in patients between the ages of 20 to 64 years, SSRI therapy was not associated with an increased risk of arrhythmias, stroke, or transient ischemic attacks in depressed patients and had a reduction in the risk of MI [25]. A study observed a significant decrease in rates of arrhythmias from 84 days after starting an SSRI [25]. Drug-drug interactions and electrolyte abnormalities in patients with pre-existing congenital cardiopathies are important causes of QTc prolongation, arrhythmias, Brugada syndrome, syncope, Torsades de pointes, and SCD [22].

In congenital long QT syndrome, mutations in ion channels, particularly hERG channels, predispose to ventricular arrhythmias and TdP increasing the risk of SCD. Genes associated with arrhythmias include SCN4B, SCN5A, CACNL1AC, KCNQ1, KCNH2, KCNE1, ANK2, ALG10, KCNJ2, KCNE2, RYR2, KCND3, KCND2, ACE, NOS1AP, CASQ2, and Rad. Mechanisms like electrophysiological disturbances, myocarditis, and myocardial ischemia play a significant role in SCD. Congenital disorders like Jervell and Lange-Nielsen syndrome and Romano-Ward syndrome result in repolarisation disturbances leading to SCD [22].

Sertraline pharmacotherapy

Multiple studies have shown that sertraline has higher efficacy among SSRIs [26]. Sertraline is a more specific and potent inhibitor of serotonin reuptake than other SSRIs, with minimal inhibitory effects on cytochromal enzymes and with no major anticholinergic side effects [26]. Sertraline has a half-life of 26 hours, and its major metabolite desmethyl-sertraline does not have any effect on serotonin reuptake leading to a favorable safety profile in overdose [27]. Genetic polymorphisms in genes regulating the HPA axis significantly affect the response to various antidepressants [19].

According to the National Institute for Health and Care Excellence (NICE) guidelines and the Cardiovascular Reduction Early Anemia Treatment Epoetin beta** (**CREATE) trial,** **sertraline should be considered a first-line SSRI when treating depression in CVD, especially in patients with recent MI [26]. Trials showed that sertraline in combination with omega-3 supplement decreased CVD risk factors [28]. SSRIs, including sertraline, inhibit platelet activity, stabilize the endothelium and decrease inflammatory marker levels; the higher platelet activation seen in depression is countered by this phenomenon [16,29]. SSRIs markedly decrease sympathetic and adrenal activity and reduce stress-hemoconcentration, which in turn reduces the risk of CVD [19]. Sertraline is safe for post-MI depression and reduces cardiac risk factors by its effects on platelet aggregation and vascular endothelium in therapeutic doses along with aspirin [19]. Sertraline benefits patients by directly countering the effects of both depression and CVD, hence its preference in post-MI depression [19,23,29]. Patients with HF and depression with associated CAD may be candidates for sertraline on the basis of CAD alone [29]. SSRIs substantially improve patient mood post-MI and have a benign cardiac side effect profile without significant adverse effects on heart rate (HR), blood pressure (BP), LVEF, or ventricular ectopy [16].

Studies have shown that SSRIs are well tolerated in >85% of patients; patients taking sertraline were observed to have significantly fewer cardiac events like MI, stroke, recurrent angina, and CHF [16]. Patients with HF have decreased HRV, increased platelet activation, and increased inflammation - all of these issues may be ameliorated by sertraline [16]. Low levels of omega-3 fatty acids (FAs) are linked with depression and increase the risk for cardiac mortality; there is evidence that omega-3 FAs reduce the incidence of SCD in high-risk cardiac patients and enhance the efficacy of SSRIs in depression [30]. Some studies have shown that SSRIs in some patients may increase the overall risk of adverse events; therefore, benefit should outweigh the risk when administering them on an individual case-by-case basis [31]. Large-scale cohort studies confirm that SSRI use decreased the risk of MI in the first year after initiation of therapy and did not increase the risk of MI in long-term usage [32].

**Figure 4:**
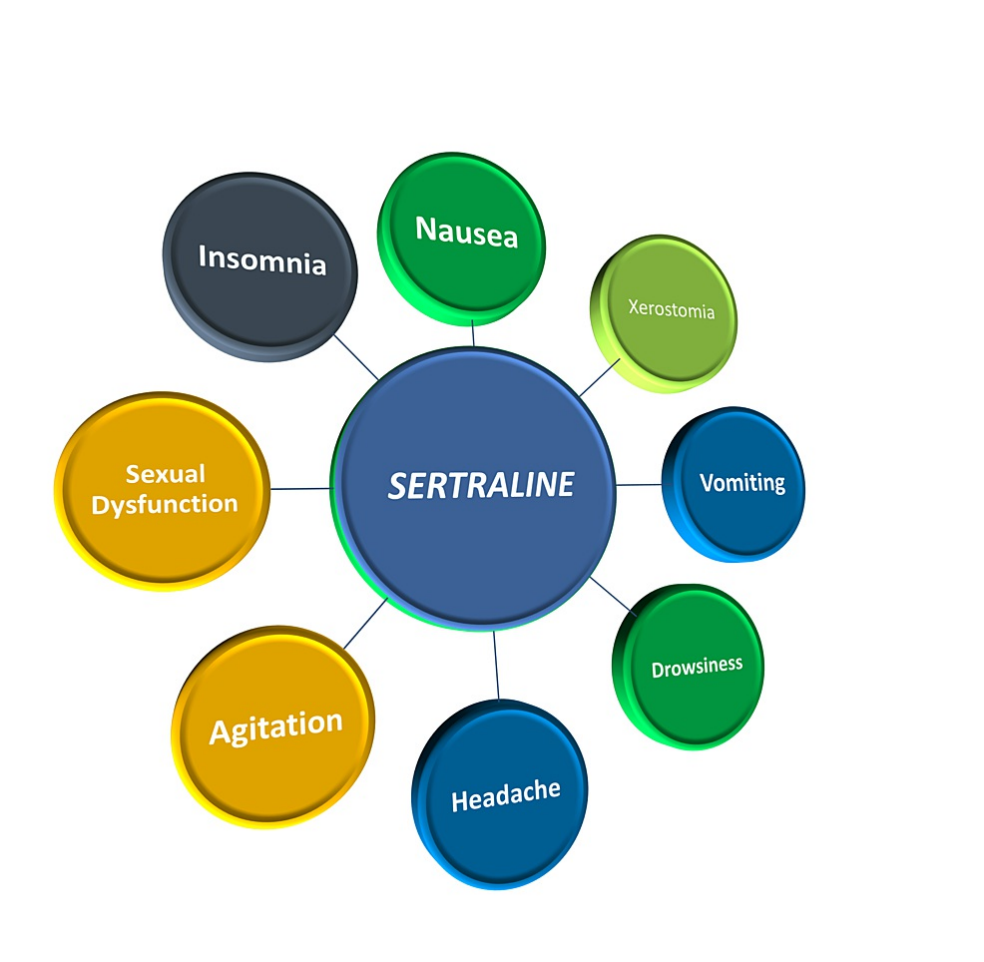
The common adverse effects of sertraline

SSRIs are the first choice of treatment in elderly patients with CVD [32]. The most extensively used medications in America are antidepressants, and more than 60% of patients have been taking antidepressants for two years or longer; women are twice as likely to be taking antidepressants than men, and SSRIs are the most commonly prescribed antidepressants. An increase in body weight, BMI, fasting glucose, total cholesterol, low-density lipoprotein (LDL), and triglyceride levels are the negative effects of SSRIs on cardiovascular health, but all these facts are also associated with food consumption which may be ameliorated with the decrease in depressive symptoms [23]. Important adverse effects of SSRIs include mild bradycardia, minimal QTc prolongation, syncope, and orthostatic hypotension [19]. The common side effects of sertraline and other SSRIs include nausea, xerostomia, vomiting, drowsiness, headache, insomnia, sexual dysfunction, agitation, and extrapyramidal symptoms like akathisia, dyskinesia, parkinsonism, and dystonia occur within the first thirty days of treatment and sertraline is one of the SSRIs frequently involved. Rashes, photosensitivity, spontaneous bruising, alopecia, urticaria, and pruritis are also associated with SSRIs. SSRIs have the highest risk of hyponatremia among all classes of antidepressants [24]. The common adverse effects of sertraline are shown in Figure 4.

Multiple trials suggested that SSRIs decrease morbidity and mortality up to 43% post-MI, even in severely depressed patients [19,23,33]. Studies show that SSRIs, including sertraline, may increase morbidity and mortality in susceptible depressed CVD patients with an increased risk of cerebrovascular disease like ischemic stroke, almost 25% to 40%, and higher rates of non-fatal MI, CHF, and unstable angina with associated increased carotid intimal-medial thickening and atherosclerosis in coronary arteries which is a strong predictor of MI risk, especially in elderly [23,25,32]. Sertraline is protective against weight gain and pre-diabetic carbohydrate metabolism changes [23]. Sertraline has been shown to decrease the odds of MI in depressed patients by its effect on increasing serotonin transporter affinity [15]. SSRIs, in combination with psychotherapy, CBT, and good social support, have been shown to give the best results as outcomes in post-MI depression [15]. Among SSRIs, sertraline is postulated to be free of cardiotoxicity and safe in treating depression in CVD patients due to its favorable effects on platelet activation, atherosclerosis, and the development of coronary artery disease. SSRIs are useful in decreasing stress and inflammation [19]. SSRI adverse effects like blurry vision, drowsiness, and sexual dysfunction are usually common in the first three months of therapy, but there have been no reports of increased bleeding with SSRI use in patients receiving antiplatelet and anticoagulation therapies for ACS. SSRIs are usually safe in drug overdoses, with insignificant QT prolongation in most cases, and rarely evoke serotonin syndrome [19].

Stress and depression are associated with immune upregulation; antidepressants downregulate the immune system negating these effects. The risk of death and non-fatal MI was significantly lower in patients taking SSRIs. Eighteen percent of all the SSRI prescriptions were for sertraline, and SSRIs make up 55% of all antidepressant prescriptions. Sertraline has one of the highest serotonin reuptake inhibition among SSRIs and may lead to abnormal bleeding and reduced platelet aggregation, especially with chronic use [19]. Many studies have shown that sertraline, in particular, is safe in post-stroke and post-MI depression. Studies have shown that SSRIs are efficacious against atherosclerosis, CAD, and depression [19]. Antidepressant effects are seen with statins, angiotensin-converting enzyme inhibitors/angiotensin receptor blockers (ACEIs/ARB), and aspirin by various mechanisms, while calcium channel blockers (CCBs), diuretics, and nitrates are associated with depression [34]. Only 30% of patients taking SSRIs are also undergoing psychotherapy [24].

SSRIs are commonly prescribed for anxiety, autism, eating disorders, premenstrual syndrome/premenstrual dysmorphic disorder, menopausal vasomotor symptoms, nociceptive pain, and gastrointestinal disorders in patients who may also be predisposed to CVD and MDD. Sertraline is an approved medication for MDD, obsessive-compulsive disorder, premenstrual dysphoric disorder (PMDD), and post-traumatic stress disorder (PTSD) and is reported to be more effective in the acute treatment of MDD than other SSRIs. Sertraline has higher rates of diarrhea than other SSRIs. Sertraline, along with other SSRIs, has a higher specificity for the serotonin transporter (SERT), thus avoiding many of the anti-histaminergic, anti-adrenergic, and anti-muscarinergic side effects of other classes of antidepressants. Sertraline has linear pharmacokinetics unlike other SSRIs, which makes it more preferable in elderly patients with comorbidities and impaired drug elimination; it is metabolized by cytochrome P450. Some studies have shown that sertraline has inhibitory effects on the cytochrome P450 system and may lead to drug-drug interactions. All SSRIs inhibit CYP2D6 but do not have a significant effect on CYP3A4 [24]. According to a study, one-third of patients with CAD were on antidepressant medications, particularly sertraline and citalopram [35]. In animal studies, sertraline has been shown to exert a strong vasodilatory effect on coronary flow (CF), and after a brief positive ionotropic phase, it has strong negative ionotropic and chronotropic effects leading to direct cardiomyocyte damage; these effects are seen in a range of 10 to 100 times the therapeutic dosage. Sertraline had no effect on nitric oxide levels in coronary vessels in any condition. The effects of SSRIs on the heart may be related to the blocking of voltage-gated L-type calcium channels and not the effects of serotonin reuptake** **[36].

Current response rates to antidepressants range from 50% to 55%. Sertraline did not negatively influence HR, HRV, BP, and LVEF in patients with MDD after hospitalization for MI or unstable angina. Sertraline is more effective at treating recurrent and severe depression in cardiac patients compared to new-onset depression. In a study comparing sertraline, placebo pill, supervised exercise, and home-based exercise, it was observed that sertraline had a 47% remission rate for MDD at four months, which was significantly higher than placebo and exercise [2]. SSRIs might increase the risk of GI bleeding and hemorrhagic stroke in cardiac patients when use concurrently with nonsteroidal anti-inflammatory drugs (NSAIDs), warfarin, or steroids and may require monitoring [2,37]. SSRIs have been reported to cause osteopenia in the elderly]. SSRIs have been reported to aggravate restless leg syndrome. Elderly patients sometimes take longer to respond to SSRIs - starting with a small dose and titrating up slowly has shown to have a lower risk of adverse effects, and treatment should be continued for at least six months and at least two years in recurrent depression. SSRIs may increase the production of brain-derived neurotrophic factor (BDNF), which promotes neurogenesis and improves neural plasticity [37].

## Conclusions

All patients post-MI must be regularly screened for depression, and sertraline is the preferred SSRI in patients with cardiovascular disease and major depression due to its favorable cardiac profile and low risk of overdose. Sertraline directly affects cardiac risk factors by its effect on serotonin and decreases depressive symptoms acting as a double-edged sword in fighting both heart disease and depression at the same time. Even though it is one of the safest antidepressants available for patients post-MI, it is always safer to screen high-risk patients with an ECG before prescribing any antidepressant. Genotyping for ion channelopathies, serotonin transporter gene mutations, congenital long QT syndromes, and cytochrome polymorphisms may also be required on a case-by-case basis.

There have been very rare instances of QT prolongation and adverse effects even with the use of sertraline, but each time, there is an associated drug-drug interaction or underlying comorbid condition that triggers the arrhythmic event. In light of the extensive use of SSRIs, especially sertraline, we would encourage increased post-marketing surveillance and large-scale observational studies as well as clinical trials to identify potential factors that may lead to adverse cardiac outcomes with sertraline use. Finally, we can conclude, based on the data in our research, that sertraline is safe and effective in treating post-MI depression and should be considered foremost among various SSRIs for the treatment of depression in heart disease.
